# Genetic heritability of age at menarche and its association with early menopause in the Korean population: a secondary data analysis

**DOI:** 10.7586/jkbns.25.041

**Published:** 2025-11-03

**Authors:** Hyerim Shin, Hye Kyung Jeon, Hae Young Yoo

**Affiliations:** 1 Department of Nursing, Graduate School of Chung-Ang University, Seoul, Korea; 2 Department of Nursing, Chung-Ang University, Seoul, Korea

**Keywords:** Menarche, Menopause, premature, Genome-wide association study, Genetic risk score

## Abstract

**Purpose:**

Age at menarche (AAM) is influenced by genetic and environmental factors and is associated with reproductive health outcomes. Therefore, understanding its genetic basis may help predict risks to women’s health. This study aimed to identify genetic variants associated with AAM in the Korean population through a genome-wide association study (GWAS) and investigate the relationship between AAM and early menopause.

**Methods:**

In total, 6,150,556 single nucleotide polymorphisms (SNPs) were generated from full genotyping data of the Health Examinee and Korea Association Resource cohorts, and analyses were conducted on 40,570 selected participants. A GWAS was performed using linear regression analysis with age and body mass index as covariates. A polygenic risk score (PRS) for AAM was constructed and categorized into quartiles, and logistic regression analysis was conducted to assess the association between the PRS for AAM and early menopause.

**Results:**

The GWAS identified 30 significant SNPs associated with AAM. The most significant SNP was rs314275 (beta = 0.0925, *p* = 2.74 × 10^−12^) in the Lin-28 homolog B (*LIN28B*) gene and the SNP with the largest effect estimate was rs67367692 (beta = 0.1238, *p* = 5.06 × 10^−8^) in the matrix metallopeptidase 12 (*MMP12*) gene. Individuals with a higher PRS for AAM showed an increased risk of early menopause.

**Conclusion:**

Genetic determinants influence AAM and early menopause in the Korean population. These findings suggest that the genetic factors contributing to late menarche may also increase the risk of early menopause, highlighting the importance of considering genetic risks in women’s health strategies.

## INTRODUCTION

Menarche, an objective and clear indicator of secondary sexual characteristics in women, is a significant indicator of sexual maturity [1]. Age at menarche (AAM) varies by race and country and has been declining globally [2-4]. AAM is influenced by intrinsic factors such as genetics and racial differences, as well as extrinsic factors such as nutritional status, socioeconomic level, stress, and their interactions [5-8]. Studies conducted on twins and their families have confirmed that genetic influence is a decisive factor in determining AAM [9].

Recent genome-wide association studies (GWAS) have been conducted to advance our understanding of the genetic factors that influence AAM. A large-scale GWAS meta-analysis using the ReproGen Consortium, involving over 87,000 people in Europe, by Elks et al. [8] identified 30 AAM genetic variants, including Lin-28 homolog B (LIN28B) on 6q21 and Long intergenic non-protein coding RNA 1505 (LINC01505) on 9q31.2, which were strongly associated with AAM and confirmed as key regulators of secondary sexual characteristics. Subsequent studies have further identified AAM genetic variants across diverse ethnic groups [10-12]. However, most of these studies have focused on Western populations, including those in Europe and the United States, while limited research has been conducted in East Asia.

AAM and age at menopause are critical components of reproductive lifespan and appear to be influenced by shared genetic and hormonal mechanisms [13,14]. Menarche that occurs earlier or later than the normal age range is known to increase the risk of hormone-related disorders, metabolic disorders, and cardiovascular diseases [15-17]. Irregularities in the reproductive cycle due to abnormal menarche timing can lead to ovarian dysfunction, hormonal imbalances, and shortened reproductive lifespan, which may affect menopause timing [18,19].

Early menopause is known as a risk factor of cardiovascular disease, osteoporosis, and hormone-related diseases, and several studies are conducted to predict and prevent its onset [20,21]. In the prediction of early menopause, various indicators such as family history, childbirth experience, smoking status, weight, and hormone levels are used [20,21]. Notably, based on research findings that genetic variants determining AAM are involved in the regulation of sex hormones and may influence the age at menopause, their potential as predictive markers for early menopause has been proposed [22,23]. While several large cohort studies have examined the general relationship between the timing of menarche and menopause, few studies have specifically investigated the association between genetic variants linked to AAM and the risk of early menopause [24,25]. Given the shared genetic and hormonal basis between menarche timing and menopause, studying genetic variants related to AAM may offer the earliest feasible indicator of reproductive aging, enabling early risk stratification and timely preventive strategies for early menopause.

Therefore, this study aimed to identify genetic variants associated with AAM in the Korean population through a GWAS and investigate the relationship between AAM and early menopause, clarifying the predictive potential of menarche-related genetic factors in women’s reproductive health.

## METHODS

### 1. Study design

This study was a secondary analysis of the existing epidemiological and genetic data from the Korean Genome and Epidemiology Study (KoGES), administered by the Korea Centers for Disease Control and Prevention.

### 2. Participants

This study included 38,400 women aged 40~76 years from 58,693 participants in the Health Examinee (HEXA) cohort and 2,877 women aged 40~69 years from 5,493 participants in the Korea Association Resource (KARE) cohort. Among them, 518 participants in the HEXA cohort and 44 participants in the KARE cohort were excluded owing to missing AAM data. Additionally, 121 and 24 participants from the HEXA and KARE cohorts, respectively, who reported an AAM of 21 years or older were excluded due to potential pathological factors and recall bias. Consequently, the study included 37,761 and 2,809 participants from the HEXA and KARE cohorts, respectively.

### 3. Genotyping and quality control

Genetic information was analyzed using DNA samples from the Korea Biobank Array (K-chip). In this study, 6,150,556 single nucleotide polymorphisms (SNPs) were generated from the full genotyping data, and analyses were conducted on 40,570 participants from the HEXA and KARE cohorts. To ensure high quality genotype data, SNP quality control (QC) procedures were performed. For the QC, SNPs were excluded if they had a minor allele frequency (MAF) < 0.05, SNP call rate < 0.95, and Hardy-Weinberg equilibrium *p* < 1 × 10^−6^. In total, 5,418,317 SNPs were retained for analysis. Data purification and analyses were conducted using PLINK version 2.0 [26].

### 4. Data collection

Data were collected from the urban-based HEXA cohort between 2004 to 2013, as well as data from the Ansan-Ansung community-based KARE cohort collected from 2001 to 2002, all of which are part of the epidemiological and genetic data provided by KoGES. Genetic data included Korean Biobank Array (K-chip) data.

### 5. Statistical analysis

The GWAS was performed using linear regression analysis, with age and body mass index (BMI) as covariates. In the discovery stage, an analysis of 37,761 participants from the HEXA cohort identified 39 significant SNPs that reached a *p* < 1 × 10^−5^, a threshold commonly used on exploratory GWAS to detective suggestive associations [27]. To enhance statistical reliability and reduce the risk of false discoveries or the Winner’s Curse, we employed a two-stage design: the identified SNPs were validated in an independent replication cohort, followed by a meta-analysis combining both cohorts. The replication stage was conducted on 2,809 participants from the KARE cohort to test whether the 39 SNPs were also replicated. Subsequently, a meta-analysis was performed to combine the GWAS results from the two cohorts and enhance statistical power. Functional mapping and annotation of genome-wide association studies (FUMA-GWAS) were used to interpret the GWAS results and generate plots [28], while dbSNP [29] and the GWAS Catalog [30] were used for functional analysis of the detected SNPs. The effects of the minor allele of significant AAM SNPs on early menopause were analyzed using PLINK version 2.0 and SPSS version 23.0 (IBM Corp., Armonk, NY, USA). Logistic regression was conducted using SPSS to calculate the odds ratios (ORs) and 95% confidence intervals (CIs) using the major allele group (C:C) as the reference. Early and late menarche were defined as age under 12 and over 15 years, respectively. Participants who reported that they had no menstruation for more than 12 months were classified as having early menopause if they experienced menopause before the age of 45 years and were categorized accordingly based on early menopause status.

Polygenic risk score (PRS) was calculated using PRSice-2 [31]. In this process, the East Asian samples from the 1000 Genomes Phase 3 were used as the linkage disequilibrium reference panel. The PRS was constructed using SNPs that reached suggestive significance (*p* < 1 × 10^−5^) in the GWAS discovery stage. It was calculated using beta values (Si), which represent the effect sizes of alleles, allele dosages (Gij), and the number of alleles for the j^th^ individual included in the PRS (Mj).


$${\mathrm{PRS}}_{j}=\Sigma (S_{i}\times G_{\mathrm{ij}})/M_{j}$$

To assess the effect of AAM PRS on early menopause, the PRS results were divided into quartiles. Logistic regression analysis was conducted using SPSS with the PRS 1^st^ quartile group as a reference. The analysis was performed for two models: one without any covariate adjustments and the other adjusted for age, BMI, and childbirth experience. These variables were selected based on their established associations with menopause timing and, in the case of childbirth experience, its role as a general reproductive indicator [24,25].

### 6. Ethical considerations

This study was a secondary analysis of publicly available, de-identified cohort data provided by KoGES and was deemed exempt from ethical review by the Institutional Review Board of Chung-Ang University (IRB No.: 1041078-202110-HR-281-01).

## RESULTS

### 1. GWAS findings

In the GWAS discovery stage conducted on the HEXA cohort, 39 significant SNPs were identified based on a *p* < 1 × 10^−5^ (Table 1). The most significant SNP was rs314275, located in the Lin-28 homolog B (LIN28B) gene (*p* = 6.56 × 10^−13^), and the SNP with the largest effect estimate was rs67367692, located in the Matrix metallopeptidase 12 (MMP12) gene (*p* = 5.06 × 10^−8^).

**Figure 1.**
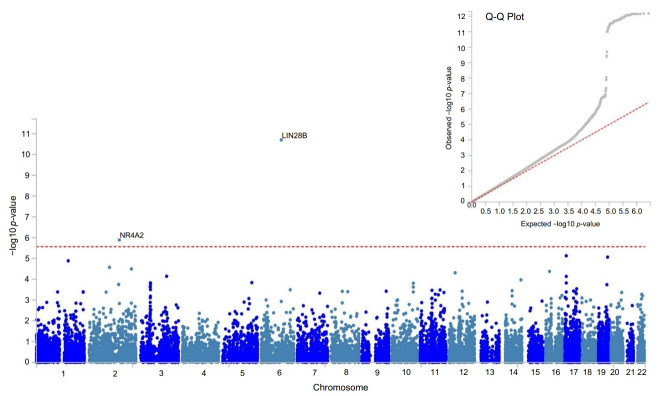
Manhattan plot and quantile-quantile (Q-Q) plot of GWAS results from the FUMA-GWAS. GWAS = Genome-wide association study; FUMA-GWAS = Functional mapping and annotation of GWAS.

Manhattan and Q-Q plots of the GWAS results are presented in Figure 1. Meta-analysis was performed on the two cohorts, resulting in 30 SNPs with *p* < 1 × 10^−5^ out of the 39 significant SNPs identified in the discovery stage (Table 2). Table 2 lists the results in ascending order of *p*-values, with the most significant SNPs identified as rs314275 (beta = 0.0925, *p* = 2.74 × 10^−12^) in the LIN28B gene, rs1150145 (beta = −0.0635, *p* = 3.30 × 10^−8^) in the Nuclear receptor 4A2 (NR4A2) gene, rs67367692 (beta = 0.1238, *p* = 5.06 × 10^−8^) in the MMP12 gene, and rs1123944 (beta = −0.0857, *p* = 6.12 × 10^−8^) in the Retinoid X receptor gamma (RXRG) gene. The beta values in Table 2 were converted into Menarche β values (in weeks) based on research by Elks et al. [8] and He et al. [7] to represent their effects on menarche timing. The results showed that rs314275 and rs67367692 were associated with delay of menarche by approximately 4.8 weeks and 6.5 weeks, respectively, whereas rs1150145 and rs1123944 were associated with advancing menarche by approximately 3.3 weeks and 4.5 weeks, respectively. The effects ranged from a maximum of 6.5 weeks to a minimum of 2.6 weeks.

Analysis of the effect of the minor allele on early menopause showed that the most significant AAM-associated SNP, rs314275, in the LIN28B gene was also linked to the risk of early menopause. Among individuals with late menarche (> 15 years), those carrying the C:T and T:T alleles had 1.15 and 1.18 times higher risk of early menopause, respectively, than those with the C:C allele (Table 3). Only individuals with early menarche (< 12 years) who carried the T:T allele showed 0.45 times reduced risk of early menopause compared with those carrying the C:C allele.

### 2. PRS-based analysis of early menopause risk

The AAM PRS was calculated based on 39 significant SNPs and categorized into quartiles for further analysis. The full list of SNPs, along with their effect sizes and genomic annotations, is provided in Table 1. To assess the risk of early menopause among AAM PRS groups, the 1^st^ PRS quartile was set as the reference group, and unadjusted OR and adjusted OR, controlling for age, BMI, and childbirth experience, were calculated (Table 4). Based on the significant results with a *p* < .05, the OR values in the 3^rd^ and 4^th^ quartiles were higher than those in the reference group after adjusting for covariates, and the risk of early menopause increased as the PRS increased.

## DISCUSSION

This study identified SNPs associated with AAM and analyzed the relationship between late AAM and early menopause, demonstrating their genetic association. A total of 30 SNPs associated with determining AAM were identified, with rs314275 in the LIN28B gene being the most significant. Among the significant SNPs, a novel SNP, rs67367692, in the MMP12 gene was identified to have the largest effect size, and it had not previously shown an association with AAM in existing studies. Analysis of the association between late AAM and early menopause confirmed that the minor allele of the LIN28B gene and higher AAM PRS groups were associated with a higher risk of early menopause.

We found rs67367692 in the MMP12 gene among the SNPs identified in the AAM GWAS result. Matrix metalloproteinases (MMPs) are zinc-dependent endopeptidases that cleave protein components of the extracellular matrix and are crucial in tissue remodeling and degradation [32]. MMPs are expressed in the human endometrium, regulated by ovarian steroid hormones, growth factors, and cytokines, and are involved in embryonic development, reproduction, menstrual processes, and the physiological stability and cyclic restricting of the endometrium [33–36]. In particular, MMP12 is a macrophage metalloelastase of the proteinase family that is expressed only during menstruation. Notably, messenger ribonucleic acid (mRNA) levels significantly increase when the concentrations of estradiol and progesterone decrease at the onset of menstruation [35,37]. Additionally, MMP12 is produced by macrophages secreted by trophoblast cells and actively participates in elastin degradation within the walls of the spiral uterine arteries [38]. Accordingly, although MMP12 is associated with hormones, reproduction, and the menstrual cycle, this is the first report of its identification as a potential determinant of AAM.

In this study, rs314275 in the LIN28B gene showed the strongest association with AAM. LIN28B, located on 6q21, is known as one of the key genes determining the timing of menarche and a genetic marker associated with pubertal growth and development [7,8,39–41]. LIN28B is an RNA-binding protein that is expressed in the hypothalamus, pituitary gland, and ovaries and is crucial in regulating the maturation of the hypothalamic–pituitary–gonadal axis [42,43]. LIN28B has an inhibitory effect on the secretion of gonadotropin-releasing hormone (GnRH) [41,44]. With a decrease in LIN28B expression during puberty, the inhibitory effect weakens, leading to increased GnRH release and the subsequent stimulation of gonadotropin (LH and FSH) secretion, ultimately increasing sex hormone levels and triggering the onset of menarche [44]. Therefore, higher mRNA levels of LIN28B in the pituitary gland are associated with late AAM. This is consistent with previous research, which supports the findings of our study on rs314275, the SNP with the strongest association in our analysis [45]. Among the genetic variants associated with LIN28B, rs7759938, rs314277 and rs1128546 have been reported across various ethnic groups, whereas rs314275, identified in this study, was discovered for the first time in a Korean cohort [8,41,46,47]. Our study also identified rs314275 in the LIN28B gene as a potential genetic factor influencing reproductive lifespan. In our SNP-based analysis, among individuals with late menarche, those carrying the C:T and T:T alleles of rs314275 had a higher risk of early menopause. Although no direct association between LIN28B and early menopause has been established, previous studies have suggested that LIN28B gene is functionally connected to estrogen signaling through hormonal regulation [48,49]. As LIN28B regulates pubertal timing via GnRH secretion, it may also contribute to downstream endocrine processes, including transition to menopause. This pathway offers a functional link between LIN28B and the regulation of reproductive timing across women’s lifespan.

Furthermore, our study revealed that individuals with a high AAM PRS have an increased genetic risk for early menopause. As a high AAM PRS is associated with late menarche [50], the risk of early menopause increases as AAM is delayed. This aligns with our SNP-based analysis—PRS is derived from the cumulative effect of multiple SNPs. Previous studies have reported that early menarche is associated with early menopause owing to reproductive factors affecting the number of ovarian cycles [24,25]. However, our findings suggest that the genetic factors associated with late menarche may also contribute to early menopause, indicating a shared genetic basis for these two traits. Therefore, in the Korean population, genetic factors associated with late menarche may increase the risk of early menopause, emphasizing the need for further research on the population-specific genetic effects on reproductive lifespan. Our study suggests that in the Korean population, genetic factors that delay menarche may simultaneously predispose individuals to early menopause, highlighting the need for further research on population-specific genetic effects in reproductive aging.

This study identified SNPs associated with AAM using large-scale cohort data from over 30,000 individuals and identified novel genetic variants specific to the Korean population. This is the first study to examine the association between late AAM and early menopause in the Korean population. By investigating the association between menarche and menopause, this study provides insights into the genetic determinants of women’s reproductive health. Nevertheless, this study had some limitations. First, as the age distribution of the study participants is in the range of 40~60 years, the long interval periods for AAM may pose a potential issue of recall bias. Second, it may not have fully accounted for other pathological, environmental, or lifestyle-related factors that could influence menopause. Third, while we focused on the functional interpretation of the LIN28B and MMP12 genes based on their statistical significance and biological relevance, other significant SNPs must be explored in future studies to provide a more comprehensive understanding of their genetic influence on AAM. Additionally, there may have been unadjusted covariates in the PRS analysis that could have significantly affected the results. Future studies are necessary to make these findings more useful by including pathological, environmental, and lifestyle-related factors and integrating results from various ethnic groups. In the context of nursing research, it is also crucial to explore how genetic information related to reproductive aging can be integrated into risk stratification, patient education, and personalized reproductive health counseling. Nursing professionals may play a key role in translating polygenic risk information into practical health guidance, especially for women at risk of early menopause. Furthermore, nursing interventions targeting modifiable lifestyle or reproductive factors can be developed to mitigate these risks, suggesting practical directions for intervention design and implementation in nursing practice.

## CONCLUSION

This study identified the genetic factors influencing AAM and examined the impact of menarche-related SNPs on early menopause. Notably, rs67367692 in MMP12 and rs314275 in LIN28B play major roles in determining AAM, and individuals carrying the minor alleles of rs314275 show a higher risk of early menopause. A higher AAM PRS is associated with an increased risk of early menopause, supporting the contribution of menarche-related genetic variants to reproductive health. These findings support the integration of genetic predispositions into women’s health management and biological nursing strategies, particularly for preventing outcomes such as early menopause, which may threaten women’s health.

## Figures and Tables

**Table 1.** SNPs Significantly Associated with Age at Menarche in the HEXA and KARE Cohorts (*p* < 1 × 10^−5^)^†^

					Discovery stage				Replication stage			
					HEXA				KARE			
CHR	POS	SNP	A1	A2	MAF	BETA	SE	*p*-value	MAF	BETA	SE	*p*-value
1	165406865	rs1123944	A	G	0.1538	−0.0849	0.0163	2.22× 10^−7^	0.1518	−0.0960	0.0608	0.1145
2	105865050	rs35697404	G	A	0.2063	−0.0680	0.0145	2.92× 10^−6^	0.2116	−0.0780	0.0546	0.1534
2	153467115	rs6731699	A	T	0.2830	0.0649	0.0130	6.87× 10^−7^	0.2818	−0.0257	0.0489	0.5991
2	153477103	rs12621715	T	C	0.4132	0.0531	0.0120	9.57× 10^−6^	0.4145	0.0005	0.0451	0.9900
2	157178819	rs1150145	T	A	0.4804	−0.0651	0.0119	4.37× 10^−8^^‡^	0.4805	−0.0405	0.0442	0.3594
2	219743874	rs3806557	A	G	0.4576	0.0553	0.0119	3.67× 10^−6^	0.4515	−0.0263	0.0445	0.5536
3	49943756	rs2293018	T	C	0.1184	0.0817	0.0182	7.67× 10^−6^	0.1157	0.0122	0.0705	0.8624
3	50520639	rs80049775	A	C	0.1257	0.0898	0.0178	4.87× 10^−7^	0.1254	0.0848	0.0679	0.2121
3	50532006	rs6807916	G	A	0.2975	0.0599	0.0129	3.74× 10^−6^	0.3033	0.0695	0.0484	0.1514
3	51581509	rs11720298	G	A	0.2942	0.0597	0.0129	4.07× 10^−6^	0.2917	0.0607	0.0484	0.2105
3	51616746	rs75629698	G	A	0.1803	−0.0789	0.0153	2.89× 10^−7^	0.1760	−0.0354	0.0576	0.5383
3	51631724	rs116883511	A	G	0.1204	0.0856	0.0181	2.36× 10^−6^	0.1216	0.0720	0.0693	0.2984
3	156791268	rs2321536	C	T	0.3463	0.0619	0.0124	6.94× 10^−7^	0.3477	−0.0471	0.0463	0.3088
5	67084776	rs55990808	T	C	0.4037	−0.0551	0.0120	4.76× 10^−6^	0.3982	−0.0451	0.0448	0.3146
5	120958166	rs28896489	G	A	0.4070	0.0562	0.0120	2.92× 10^−6^	0.4068	0.0504	0.0450	0.2631
6	19161875	rs12528805	G	A	0.3012	0.0569	0.0128	9.12× 10^−6^	0.3015	0.0596	0.0484	0.2181
6	105358192	rs6902789	A	G	0.2097	0.0829	0.0145	1.28× 10^−8^^‡^	0.2123	−0.0392	0.0543	0.4701
6	105408966	rs314275	T	C	0.2514	0.0983	0.0136	6.56× 10^−13^^‡^	0.2472	0.0078	0.0519	0.8791
7	49351293	rs58752316	C	A	0.1840	0.0725	0.0152	1.94× 10^−6^	0.1863	−0.0706	0.0559	0.2070
7	77638859	rs2960460	C	T	0.3277	−0.0558	0.0126	9.72× 10^−6^	0.3195	−0.0035	0.0473	0.9406
8	135673934	rs28719631	G	A	0.3554	−0.0584	0.0123	2.19× 10^−6^	0.3600	−0.0158	0.0461	0.7320
11	43978258	rs517465	C	T	0.4476	−0.0632	0.0119	1.10× 10^−7^	0.4397	0.0089	0.0455	0.8449
11	102718695	rs662558	C	T	0.0950	0.0905	0.0202	7.48× 10^−6^	0.0882	0.0533	0.0788	0.4986
11	102761546	rs67367692	T	C	0.0680	0.1274	0.0234	5.49× 10^−8^	0.0623	0.0679	0.0926	0.4633
11	133523968	rs76358860	T	C	0.2115	−0.0666	0.0144	4.11× 10^−6^	0.2058	0.0073	0.0553	0.8947
13	102874124	rs9554859	A	T	0.1813	−0.0704	0.0153	4.68× 10^−6^	0.1713	−0.0347	0.0588	0.5552
14	101148235	rs10137545	G	A	0.1072	−0.0846	0.0191	9.66× 10^−6^	0.1050	−0.0981	0.0744	0.1874
14	101187072	rs76646633	G	A	0.0785	−0.0998	0.0219	5.44× 10^−6^	0.0791	−0.0891	0.0834	0.2852
16	20302513	rs4238591	A	G	0.3812	0.0543	0.0122	9.15× 10^−6^	0.3791	−0.0012	0.0452	0.9789
16	20302966	rs4238592	C	T	0.1586	0.0740	0.0162	4.88× 10^−6^	0.1600	−0.0921	0.0606	0.1287
16	79608424	rs12447260	C	T	0.2210	−0.0727	0.0141	2.91× 10^−7^	0.2235	−0.0096	0.0541	0.8589
16	84421528	rs11643260	C	G	0.4494	0.0570	0.0118	1.54× 10^−6^	0.4527	0.0147	0.0444	0.7404
17	75492785	rs11652286	A	G	0.2070	−0.0668	0.0145	4.46× 10^−6^	0.2037	0.0258	0.0543	0.6341
19	47563418	rs12150914	C	T	0.2178	−0.0684	0.0143	1.71× 10^−6^	0.2274	−0.0103	0.0542	0.8489
20	17091951	rs2211254	T	C	0.1585	0.0838	0.0161	2.18× 10^−7^	0.1614	0.0326	0.0606	0.5903
20	17100450	rs1101145	T	C	0.2125	0.0762	0.0144	1.25× 10^−7^	0.2121	0.0063	0.0549	0.9084
20	17126278	rs1934894	G	A	0.3475	0.0603	0.0124	1.27× 10^−6^	0.3517	0.0794	0.0466	0.0889
20	40156236	rs6129860	T	C	0.2956	0.0574	0.0129	9.24× 10^−6^	0.2963	−0.0177	0.0487	0.7167
20	40269524	rs145579032	G	A	0.0976	0.0882	0.0199	9.66× 10^−6^	0.0956	0.0454	0.0740	0.5398

HEXA = Health Examinee; KARE = Korea Association Resource; CHR = Chromosome; POS = Position; SNP = Single nucleotide polymorphism; A1 = Minor allele; A2 = Major allele; MAF = Minor allele frequency; SE = Standard error.

† MAF, BETA, SE, and *p*-value are presented to four decimal places for precision; *p*-values < .001 are reported in scientific notation.

‡ Statistically significant at *p* < 5 × 10^−8^.

**Table 2.** Summary Statistics from a Meta-analysis of GWAS (*p* < 1 × 10^−5^)^†^

SNP	CHR	POS	Gene	Function	A1	A2	Beta^‡^	Menarche β^§^ (weeks per allele)	*p*-value
rs314275	6	105408966	*LIN28B*	Intronic	T	C	0.0925	4.8	2.74×10^−12^
rs1150145	2	157178819	*NR4A2*	Intergenic	T	A	−0.0635	−3.3	3.30×10^−8^
rs67367692	11	102761546	MMP12	Intergenic	T	C	0.1238	6.5	5.06×10^−8^
rs1123944	1	165406865	*RXRG*	Intronic	A	G	−0.0857	−4.5	6.12×10^−8^
rs6902789	6	105358192	*HACE1*	Intergenic	A	G	0.0747	3.9	1.09×10^−7^
rs80049775	3	50520639	*CACNA2D2*	Intronic	A	C	0.0896	4.7	2.17×10^−7^
rs2211254	20	17091951	*PCSK2*	Intergenic	T	C	0.0805	4.2	2.64×10^−7^
rs1101145	20	17100450	*PCSK2*	Intergenic	T	C	0.0718	3.7	2.72×10^−7^
rs1934894	20	17126278	*PCSK2*	Intergenic	G	A	0.0616	3.2	3.04×10^−7^
rs75629698	3	51616746	*RAD54L2*	Intronic	G	A	−0.0760	−4.0	3.10×10^−7^
rs517465	11	43978258	*ACCSL*	Intergenic	C	T	−0.0586	−3.1	3.61×10^−7^
rs12447260	16	79608424	*WWOX*	Intergenic	C	T	−0.0687	−3.6	5.45×10^−7^
rs35697404	2	105865050	*GPR45*	Intergenic	G	A	−0.0687	−3.6	1.02×10^−6^
rs6807916	3	50532006	*CACNA2D2*	Intronic	G	A	0.0606	3.2	1.30×10^−6^
rs116883511	3	51631724	*RAD54L2*	Intronic	A	G	0.0848	4.4	1.37×10^−6^
rs28896489	5	120958166	*FTMT*	Intergenic	G	A	0.0559	2.9	1.53×10^−6^
rs11720298	3	51581509	*RAD54L2*	Intronic	G	A	0.0598	3.1	1.79×10^−6^
rs11643260	16	84421528	*ATP2C2*	Intronic	C	G	0.0542	2.8	2.25×10^−6^
rs55990808	5	67084776	*CD180*	Intergenic	T	C	−0.0544	−2.8	2.89×10^−6^
rs12150914	19	47563418	*TMEM160, ZC3H4*	Intergenic	C	T	−0.0647	−3.4	2.94×10^−6^
rs76646633	14	101187072	*DLK1*	Intronic	G	A	−0.0991	−5.2	3.00×10^−6^
rs28719631	8	135673934	*ZFAT*	Intronic	G	A	−0.0556	−2.9	3.12×10^−6^
rs6731699	2	153467115	*FMNL2*	Intronic	A	T	0.0589	3.1	3.14×10^−6^
rs10137545	14	101148235	*DLK1*	Intergenic	G	A	−0.0855	−4.5	3.93×10^−6^
rs12528805	6	19161875	None	-	G	A	0.0571	3.0	4.14×10^−6^
rs9554859	13	102874124	*FGF14*	Intronic	A	T	−0.0681	−3.6	4.67×10^−6^
rs2321536	3	156791268	*LEKR1*	Intergenic	C	T	0.0546	2.8	5.96×10^−6^
rs662558	11	102718695	*MMP3,MMP12*	Intergenic	C	T	0.0882	4.6	6.54×10^−6^
rs145579032	20	40269524	None	-	G	A	0.0853	4.4	9.34×10^−6^
rs76358860	11	133523968	*OPCML*	Intergenic	T	C	−0.0619	−3.2	9.76×10^−6^

GWAS = Genome-wide association study; SNP = Single nucleotide polymorphism; CHR = Chromosome; POS = Position; A1 = Minor allele; A2 = Major allele.

† Beta is presented to four decimal places, and *p*-values < .001 are reported in scientific notation for precision;

‡ Beta refers to the mean change in age at menarche in years per copy of the minor SNP allele;

§ Menarche β represents the beta value converted from years to weeks.

**Table 3.** Effects of the Minor Allele of a Significant SNP Associated with Early Menopause

rs314275 C>T (*LIN28B*)				
Carriers		C:C (n = 21,030)	C:T (n = 14,399)	T:T (n = 2,332)
Early AAM (< 12 years) (n = 307)	OR	1	0.98	0.45
	95% CI		0.78~1.24	0.23~0.89
	*p*-value	.068	.892	.021
Late AAM (> 15 years) (n = 14,574)	OR	1	1.15	1.18
	95% CI		1.10~1.20	1.08~1.29
	*p*-value	< .001	< .001	< .001

SNP = Single-nucleotide polymorphism; *LIN28B* = Lin-28 homolog B; AAM = Age at menarche; OR = Odds ratio; CI = Confidence interval.

**Table 4.** Odds Ratio for Early Menopause by Age According to Menarche Polygenic Risk Score Quartiles

	PRS group	Unadjusted	95% CI		*p*-value	Adjusted^†^	95% CI		*p*-value
		OR	Lower	Upper		OR	Lower	Upper	
Early menopause	1^st^ quartile (n=8,870)	1			.060	1			.096
	2^nd^ quartile (n=9,994)	1.11	0.99	1.24	.071	1.11	1.00	1.24	.060
	3^rd^ quartile (n=9,388)	1.15	1.03	1.28	.015	1.13^‡^	1.01	1.27	.030
	4^th^ quartile (n=9,509)	1.14	1.02	1.27	.020	1.13^‡^	1.01	1.27	.028

PRS = Polygenic risk score; OR = Odds ratio; CI = Confidence interval.

† Adjusted for age, body mass index, and child birth experience;

‡ Exact adjusted OR before rounding: 3^rd^ quartile=1.133; 4^th^ quartile=1.134.

## Data Availability

The data used in this study are available after obtaining permission from the National Biobank of Korea from the National Human Resources Bank of Korea, the Center for Disease Control and Prevention, Republic of Korea (https://biobank.nih.go.kr/eng/cmm/main/mainPage.do).
